# Geographic analysis of latent tuberculosis screening: A health system approach

**DOI:** 10.1371/journal.pone.0242055

**Published:** 2020-11-09

**Authors:** John P. Bonnewell, Laura Farrow, Kristen V. Dicks, Gary M. Cox, Jason E. Stout

**Affiliations:** Division of Infectious Diseases, Department of Medicine, Duke University Medical Center, Durham, North Carolina, United States of America; Agencia de Salut Publica de Barcelona, SPAIN

## Abstract

**Background:**

Novel approaches are required to better focus latent tuberculosis infection (LTBI) efforts in low-prevalence regions. Geographic information systems, used within large health systems, may provide one such approach.

**Methods:**

A retrospective, cross-sectional design was used to integrate US Census and Duke Health System data between January 1, 2010 and October 31, 2017 and examine the relationships between LTBI screening and population tuberculosis risk (assessed using the surrogate measure of proportion of persons born in tuberculosis-endemic regions) by census tract.

**Results:**

The median proportion of Duke patients screened per census tract was 0.01 (range 0–0.1, interquartile range 0.01–0.03). The proportion of Duke patients screened within a census tract significantly but weakly correlated with the population risk. Furthermore, patients residing in census tracts with higher population tuberculosis risk were more likely to be screened with TST than with an IGRA (p<0.001).

**Conclusion:**

The weak correlation between patient proportion screened for LTBI and our surrogate marker of population tuberculosis risk suggests that LTBI screening efforts should be better targeted. This type of geography-based analysis may serve as an easily obtainable benchmark for LTBI screening in health systems with low tuberculosis prevalence.

## Introduction

Tuberculosis (TB) elimination in low-prevalence areas increasingly relies on targeted testing and treatment of latent tuberculosis infection (LTBI) [1]. The United States (US) illustrates the challenge associated with LTBI testing and treatment: despite a continued decrease in new cases each year, with about nine thousand new cases in 2018, the current rate of decline makes TB elimination unlikely to occur by the end of the 21^st^ century [2, 3]. A major component of this trend has been the increasing proportion of foreign-born cases, 70% of the 2018 total, in whom the rate is fourteen times that of US-born cases (14.2 for foreign-born compared with 1.0 US-born per 100,000 in 2018) [2]. Over 80% of active TB in foreign-born persons residing in the US arises from reactivation of LTBI [4], which may occur many years after immigration; 46% of the 2018 total received a TB diagnosis ≥10 years after arrival to the US. Targeted LTBI testing and treatment of foreign-born persons has been demonstrated to be cost-effective, particularly using the interferon-gamma release assay (IGRA) for screening [5, 6]. A study comparing TB screening models in California supported a scaled-up targeted testing approach for foreign-born persons; in particular, a one-time testing strategy was deemed cost-effective in this population [7]. These models, however, rely on appropriate testing by providers, which in higher-risk foreign-born persons—those from low- and middle-income countries—should generally be IGRAs given high rates of Bacillus Calmette-Guérin (BCG) vaccination in this population [8].

A targeted screening strategy can face a challenge in that foreign-born persons access healthcare less frequently than the US-born [9, 10]. An approach to counter this problem is the use of geographic information systems (GIS), a method that uses geospatial data to map and analyze important disease-related factors, such as disease clustering. For TB, this method can take advantage of the tendency for foreign-born persons to cluster in certain areas, often within communities in and around urban centers. A number of studies to date have demonstrated that mapping of TB cases can identify areas of increased risk for purposes of targeted testing [11–14]. The GIS-based targeted testing approach of Moonan, et al. in Texas was able to identify 1 case of LTBI per 5 people screened and 1 active TB case per 84 screened, which was a very high-yield approach [12].

Hospital systems and public health officials can capitalize on GIS analysis to determine adequacy of provider screening and identify areas of increased geographic risk for disease for purposes of resource allocation. The objectives of this study were (1) to describe the trends in LTBI screening within the Duke University Health System in North Carolina and (2) to evaluate the relationship between the geographic risk of LTBI, using the risk surrogate of proportion of foreign-born persons from TB-endemic areas residing in a given area, and the proportion of patients screened for LTBI in that area.

## Methods

### Study population

The study was conducted within the Duke University Health System (DUHS) in North Carolina. The health system includes Duke University Medical Center, a 957-bed academic tertiary care hospital in Durham, in addition to two other regional inpatient facilities and numerous outpatient clinics across the state. The health system serves approximately 1.7 million people. Prior to 2016, IGRAs were sent out to an external laboratory, but in mid-2016 DUHS began performing these tests at an in-house laboratory.

North Carolina had a rate of 2.1 active TB cases per 100,000 population in 2017 (213 new cases), lower than the national average and ranking 21^st^ in the United States [15]. The state has followed the national trend of a decline in progress toward TB elimination targets, with the case rate essentially unchanged since 2012. The percentage of foreign-born cases has also increased, from 44% in 2011 to 56% in 2017 [16]. New cases primarily occur in immigrants from five countries of origin, all with national universal BCG vaccination policies—23% from Mexico, 22% India, 7% Vietnam, 7% Philippines, and 7% Honduras [16].

### Study design

We performed a retrospective cross-sectional study of all patients seen within DUHS from January 1, 2010 to October 31, 2017. Demographic and LTBI screening data were extracted from the electronic health record via the DEDUCE query tool [17]. As data on LTBI screening from other health systems was not available, we used the proportion of DUHS patients seen within the tract as a proxy for population screening. Census tracts with fewer than 20 DUHS patients and/or outside of North Carolina were excluded from the analysis to reduce referral bias. We also examined a threshold of 100 DUHS patients per census tract and obtained substantively the same results, so used the 20-patient threshold for the analysis. GIS data was obtained by using patient home addresses, which were geocoded and mapped to census tracts. Demographic summary data for each census tract was obtained from the American Community Survey (ACS) 2012–2016 dataset, which provides a single period estimate of demographic characteristics for the 5-year period; the 5-year estimates were used because they are considered statistically more reliable for small geographic areas [18]. Data on the Social Deprivation Index, a composite measure of social deprivation, were also obtained by census tract [19, 20]. We defined our population of interest, higher-risk foreign-born persons, as those born in Africa, Asia, or Latin America. The DUHS electronic health record does not routinely obtain data on country of birth, and thus we used the ACS data to determine the proportion of higher-risk foreign-born by census tract. LTBI screening was identified using the Common Procedural Terminology (CPT) codes for the tuberculin skin test (TST; code 86580) and IGRA (QuantiFERON Gold in-tube is used in DUHS; code 86480) [21].

### Statistical analysis

Medians and interquartile ranges were used to describe continuous variables, and proportions were used for categorical variables. Time trends in testing were assessed using linear regression. Pearson correlation coefficients and a single bivariable linear regression model were used to assess for relationships between census tract characteristics and proportion of DUHS patients residing within each census tract for whom a TB screening test was performed. Unconditional logistic regression using a quasibinomial error distribution to correct for overdispersion was used to examine the relationship between the likelihood to receive a TST (versus an IGRA) by census tract characteristics. All statistical analysis was performed using R, version 3.6.1 [22].

### Ethics statement

The study was approved by the Duke University Institutional Review Board (Pro00089252). The requirement for informed consent was waived by the Duke Institutional Review Board as this was a minimal risk study using preexisting data.

## Results

Of 1.68 million patients seen at DUHS, 1.28 million (76%) could be mapped to a census tract within North Carolina. North Carolina includes 2195 census tracts, of which 1366 contained at least one DUHS patient during the study period. Of these, 1351 (62% of the total) contained at least 20 DUHS patients and were considered for analysis. These tracts included 1,216,900 DUHS patients, with an estimated underlying population of 6,778,882. The percentage of higher-risk foreign-born within these tracts ranged from 0–44.7% (median 6%, IQR 3.1–10.4%). To ascertain how well DUHS patients represented the underlying census tract populations, we examined the correlations between demographic features of DUHS patients residing in a census tract and the entire census tract population for three demographic variables: proportion of Asian race, proportion of Black race, and proportion of Hispanic ethnicity. The correlation coefficients were 0.90 for Asian race, 0.95 for Black race, and 0.62 for Hispanic ethnicity. The slopes of the best-fit lines were 1.27 for Asian race, 1.01 for Black race, and 1.41 for Hispanic ethnicity, suggesting that Asians and Hispanics were proportionately underrepresented in DUHS patients relative to the underlying population. Table 1 summarizes the differences between census tracts included and excluded from analysis.

**Table 1:** Demographic summary of North Carolina census tracts that were included versus excluded from analysis.

	Excluded (N = 844), mean (SD)^a^	Included (N = 1351), mean (SD)	p-value
Population	3725 (1652)	4960 (2049)	<0.001
Social Disadvantage Index	56.3 (26.7)	54.5 (27.6)	0.122
Percent Black	18% (21)	23% (21)	<0.001
Percent Hispanic	8% (9)	9% (8)	0.329
Percent foreign-born	7% (7)	8% (6)	0.002

^a^SD is standard deviation.

**Fig 1:**
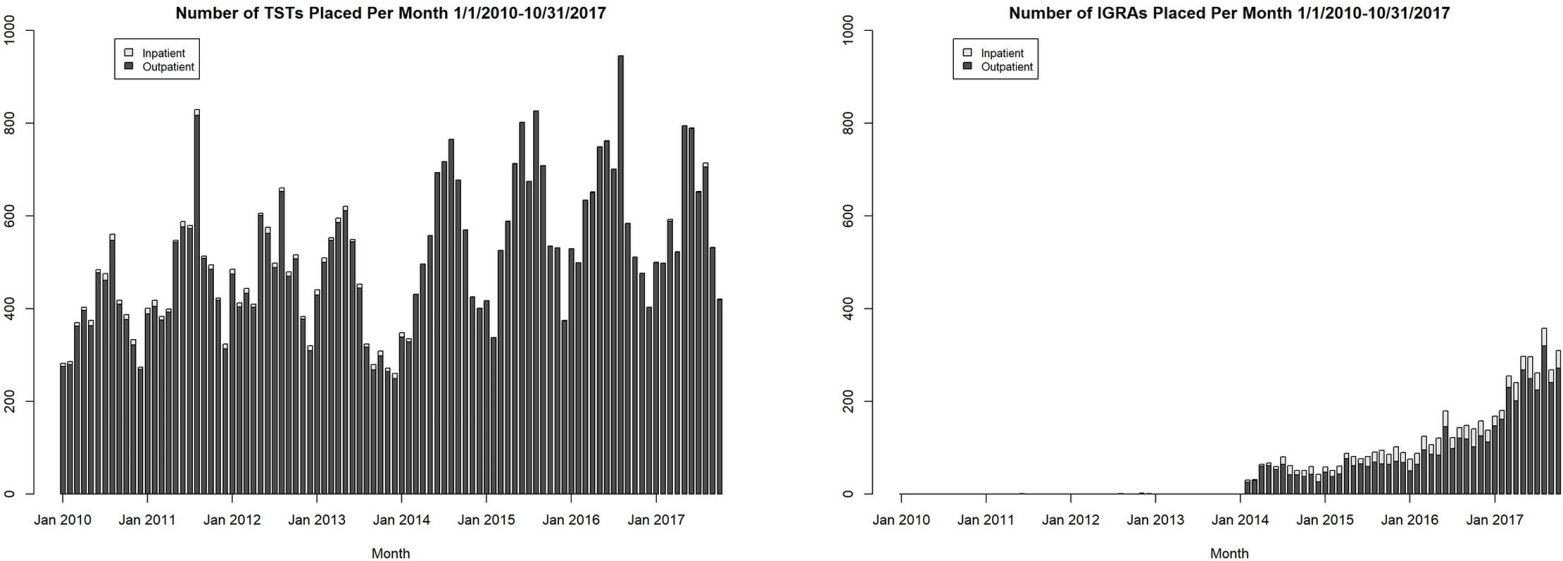
Number of TSTs and IGRAs placed per month in the Duke University Health System, January 1, 2010 to October 31, 2017.

**Fig 2:**
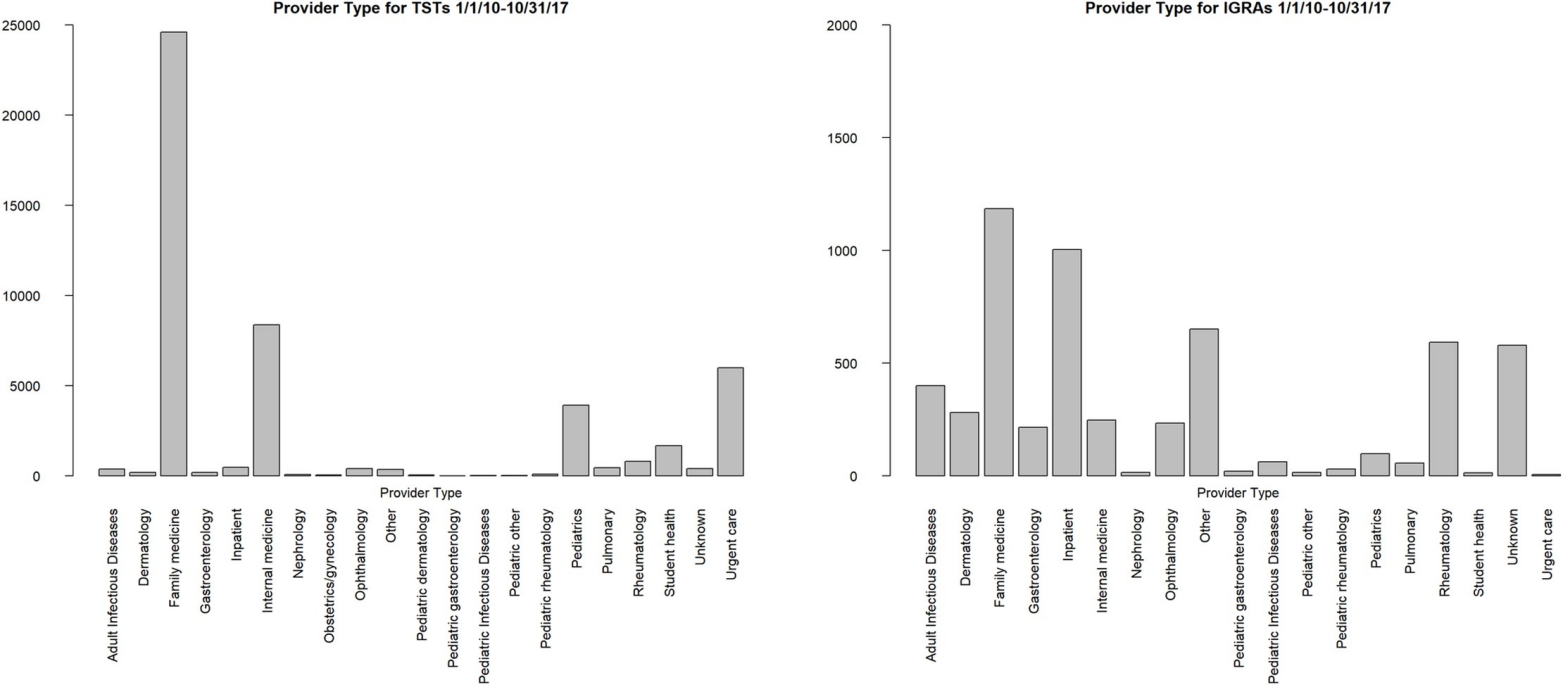
Provider type ordering TSTs and IGRAs in the Duke University Health System, January 1, 2010 to October 31, 2017.

A total of 48,399 TSTs (on 36,807 patients) and 5,728 IGRAs (on 5,350 patients) were performed at DUHS during the study period. Both TST and IGRA testing increased steadily during the study period (p<0.001 for trend for both tests), with significant cyclical variation over the year for TSTs (presumably due to occupational testing) but not for IGRAs (Fig 1). While the vast majority of TSTs were ordered by primary care (family medicine, internal medicine, or pediatrics) physicians, subspecialists (dermatology, gastroenterology, ophthalmology, and rheumatology) accounted for a larger proportion of IGRAs ordered (Fig 2).

**Fig 3:**
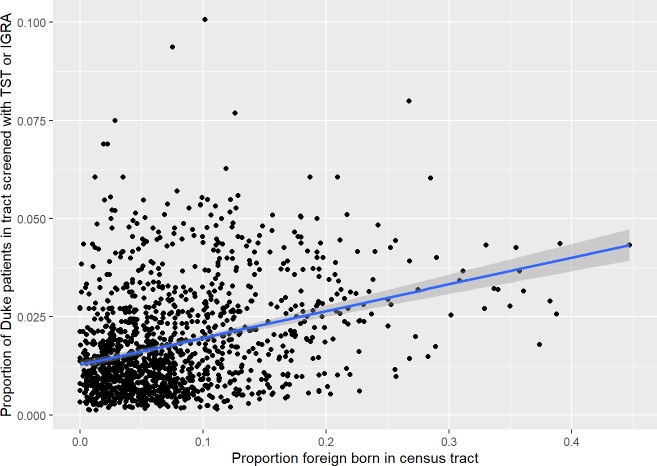
Regression model for proportion of higher-risk foreign-born living in a census tract and percentage of Duke University Health System patients living in that tract screened with TST or IGRA, January 1, 2010 to October 31, 2017.

Census tracts with a greater proportion of higher-risk foreign-born were found to be more likely to contain a higher proportion of DUHS patients screened for LTBI with TST or IGRA; however, the positive correlation was weak (Fig 3, slope = 0.068 (standard error 0.005), r^2^ = 0.105, p<0.001). There was no significant correlation between either the proportion of persons living below the poverty line in a census tract or the Social Disadvantage Index in that census tract (p>0.5 for both associations). Regarding choice of testing by providers, patients residing in tracts with a higher proportion of higher-risk foreign-born persons were less likely to be tested with an IGRA (as opposed to a TST; odds ratio 0.41, 95% confidence interval 0.19–0.87).

## Discussion

Our study proposes a novel, geography-based measure to assess how well health systems are targeting LTBI screening in low-prevalence areas. This measure integrates easily obtainable public data with administrative data that could be obtained from many large health systems in low-prevalence, higher-income countries. It also provides a method to compare targeting across different types of health systems; it would be particularly interesting, for example, to examine the same relationship in public health clinics (which serve a different population in our region and are more focused on TB control). Using the proportion of health system patients in a given geographic location partially compensates for the lack of population-based data, which often is not available given multiple health systems serving the same geographic area. Not surprisingly, we demonstrated that LTBI screening in our health system is not well-targeted to persons at higher LTBI risk (i.e., higher-risk foreign-born), as demonstrated by our proxy measure of living in a census tract with a higher proportion of higher-risk foreign-born persons. There was also no significant relationship between social disadvantage in a census tract and the proportion of our patients screened for LTBI. This may well reflect an appropriate decision as in the low-incidence US setting, poverty may independently contribute less to LTBI risk than foreign birth [23].

While the vast majority of the DUHS patient population were screened for LTBI with TST from 2010–2017, the use of IGRAs has rapidly increased since 2014. This likely reflects implementation of recent recommendations for IGRA use in the US and increasing test availability within our health system. Despite initial recommendations on IGRA use by the Centers for Disease Control and Prevention (CDC) in 2010 and a subsequent preference for IGRAs in a 2016 Infectious Diseases Society of America statement [24, 25], IGRA uptake by providers and institutions has likely been limited by high costs, lack of availability, or lack of familiarity with the cross-reactivity of TSTs and BCG antigens. Furthermore, our analysis suggests that the patient populations being tested with IGRAs may not be at high risk for LTBI; IGRA testing was relatively lower in census tracts with higher proportions of high-risk foreign-born persons. Furthermore, IGRAs were disproportionately ordered by subspecialists such as gastroenterologists and rheumatologists compared with TSTs, suggesting that much of the IGRA testing performed was directed at persons about to start biologic or other immunosuppressive therapy, who are often at low epidemiologic risk of LTBI.

There are several means by which our data could be used to make improvements within our health system. First, given the suggestion that our health system clinicians do not appear to be recognizing and prioritizing screening for those highest risk for TB, and are using the less-preferred TST rather than an IGRA for screening, there is significant opportunity for provider education and ongoing training. More importantly, recognizing census tracts with a higher proportion of foreign-born and low screening within the health system could lead to screening drives within either local DUHS facilities or as community outreach. Prior GIS-based studies have demonstrated the utility of geographically-targeted screening for active TB in certain settings [12, 14].

This study has several limitations. First, we used DUHS patients as a proxy for population screening. This may introduce bias, as our patient population may not be fully representative of the population of each census tract, and this may also vary by census tract. LTBI screening rates may be lower in the general population than in DUHS patients, particularly in foreign-born populations, given those outside our health system may have a lower propensity for seeking healthcare altogether. In addition, we were unable to directly assess if the higher-risk foreign-born DUHS patients are being screened given lack of data in our electronic medical record on country of birth. This limitation certainly raises the potential for ecologic bias, and future studies that examine individual patient data will be useful to better understand the utility of our approach. However, another limitation for this study is that we did not have actual information on test results for most patients, primarily due to unavailability of TST results in an electronically accessible format. Finally, occupational testing accounted for an unknown proportion of tests performed during the study period, and such testing may have potentially biased our results by inflating testing rates in census tracts containing large proportions of healthcare workers.

Despite these limitations, a geographic information systems-based approach to LTBI screening as outlined here has significant advantages for public health practice. A recent analysis suggested that screening only people with a strong World Health Organization screening recommendation would prevent less than 5% of incident tuberculosis cases, while testing immigrants from high-incidence countries could prevent a much larger proportion of cases [26, 27]. Given the enormity of the task, tools such as geographic information systems will be essential to efficiently identifying and screening people at risk for LTBI.

## Conclusions

Geographic analysis using readily available data was able to better understand the relationships between LTBI testing and population LTBI risk, and to point out areas for improvement in our health system. Similar efforts will be required to efficiently focus LTBI screening on populations who might benefit from testing and treatment.
